# Supplementary figures and images for: A suboptimal OCT4-SOX2 binding site facilitates the naïve-state specific function of a Klf4 enhancer
Source: PLoS One. 2024 Sep 30;19(9):e0311120. doi: 10.1371/journal.pone.0311120 (PMC11441684; doi:10.1371/journal.pone.0311120)

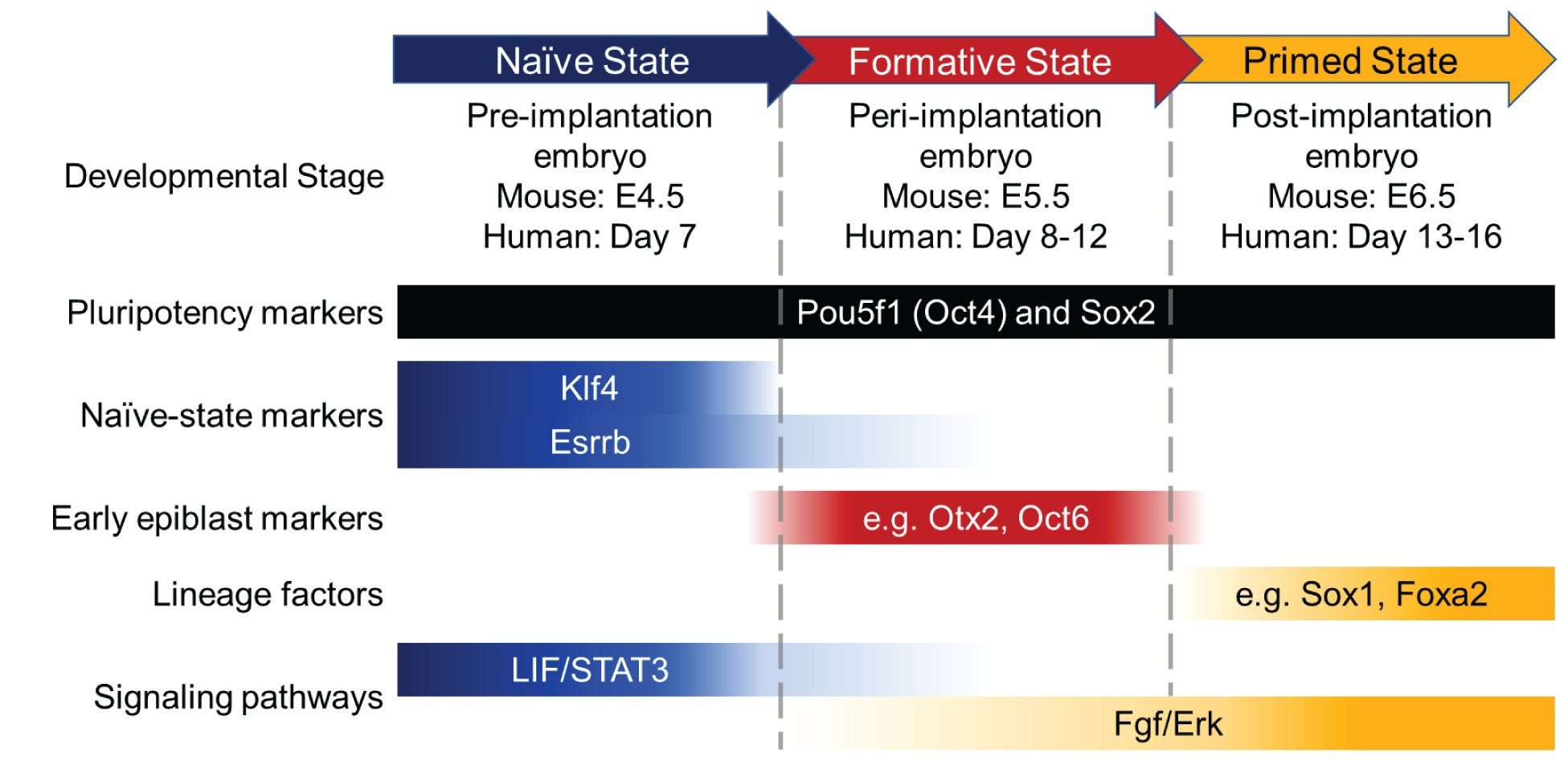

Supplement: S1 Fig — Diagram outlines how the three states of pluripotency correspond to developmental stages in mouse and human embryos. Markers and signaling pathways for each state are represented by labeled boxes, with gradients to indicate relative levels. (TIF) [file pone.0311120.s001.tif]

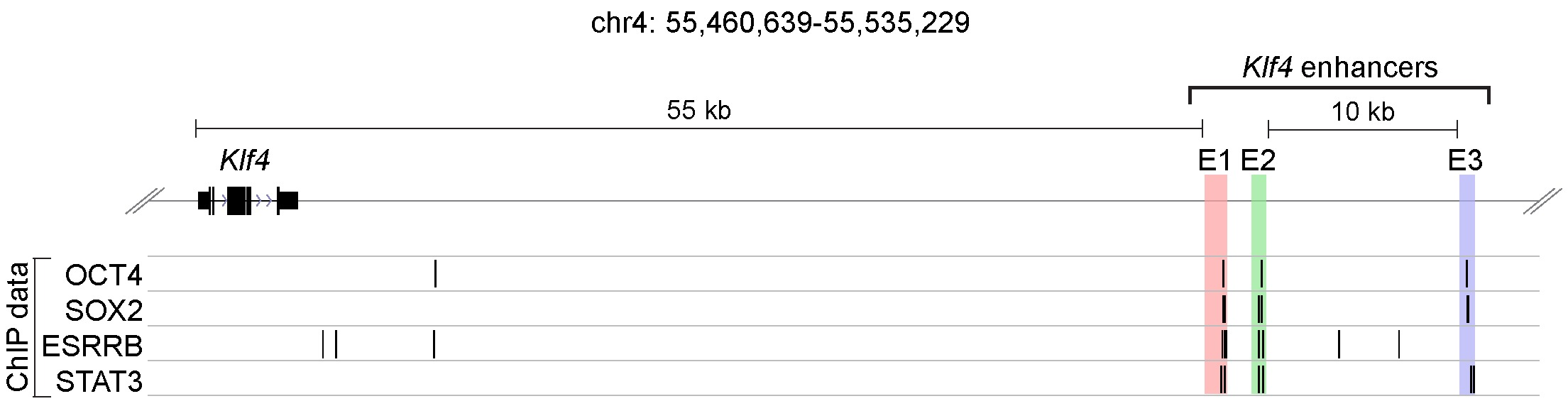

Supplement: S2 Fig — (upper) Schematic diagram of the Klf4 locus, located at chromosome 4: 55,460,639–55,535,229 (mm10) in the Mus. musculus genome. The three Klf4 enhancers (E1, E2, and E3) are located 55–75 kb downstream of the transcription start site and are highlighted in light red, green, and blue, respectively. (lower) Localization of OCT4, SOX2, ESRRB, and STAT3 across the Klf4 locus. ChIP-seq or ChIP-exo data for OCT4 [73], SOX2 [74], ESRRB [28], and STAT3 [28] were uploaded onto the UCSC genome browser and viewed on the M. musculus mm10 genome. Black bars represent locations of peaks. (TIF) [file pone.0311120.s002.tif]

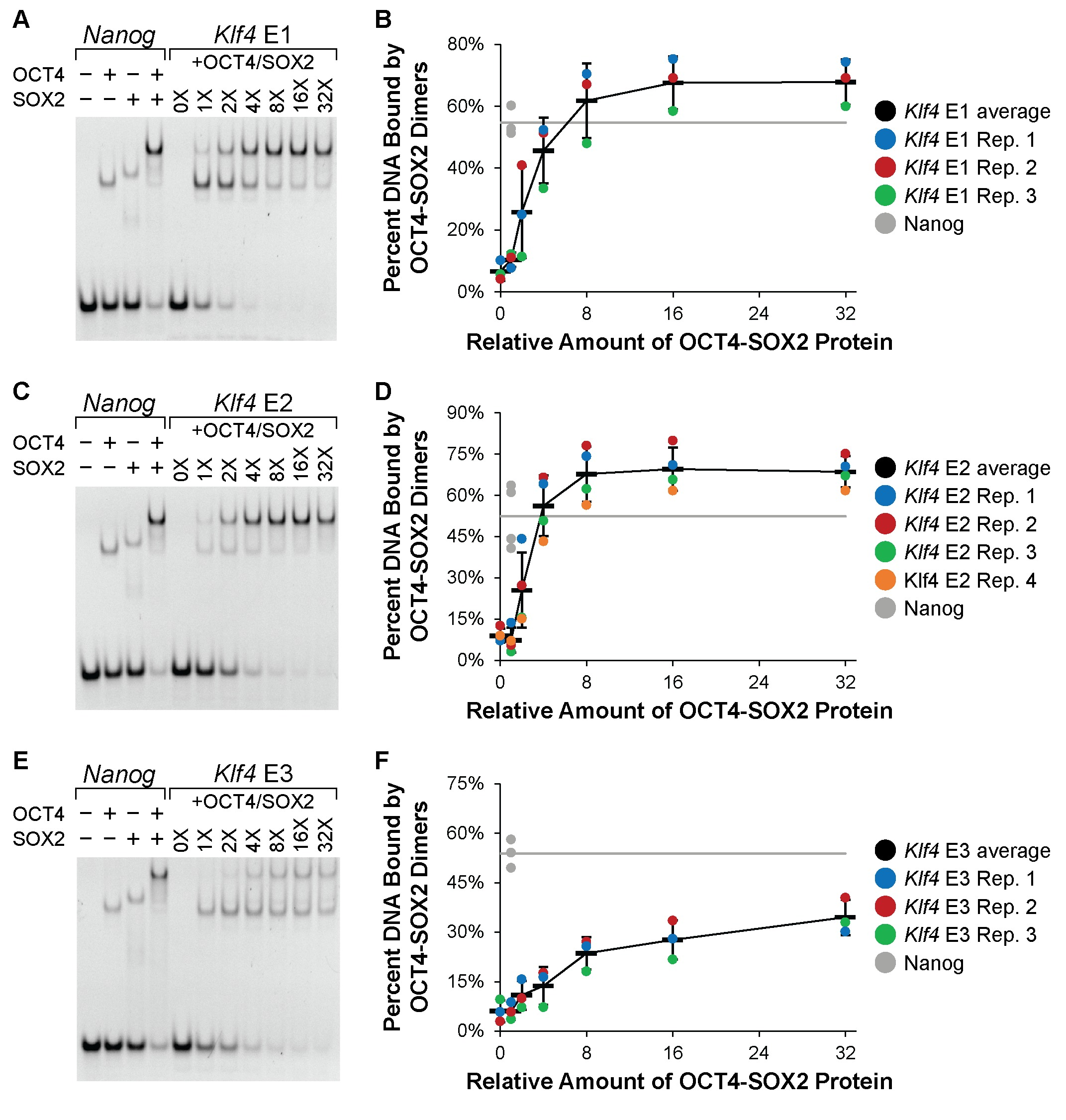

Supplement: S3 Fig — (A, C, E) Electrophoretic mobility shift assays were performed with Cy5-labeled DNA probes containing the OCT4-SOX2 composite motif (OS site) from the Klf4 enhancer E1 (A), enhancer E2 (C), or enhancer E3 (E). Binding reactions contained 2 nM of Cy5-labeled DNA probe. A dilution series of OCT4 and SOX2 was employed, in which "1X" is 19 nM OCT4 and 16 nM SOX2. A probe with the OS site from the Nanog enhancer was employed as a control. At least three replicate experiments were performed, and representative images are shown. (B, D, F) Quantitative analysis of EMSA data to assess OCT4-SOX2 binding to OS sites from Klf4 enhancer E1 (B), enhancer E2 (D), and enhancer E3 (F). The % DNA bound by OCT4-SOX2 dimers (amount of signal for the DNA-OCT4-SOX2 band/total DNA signal in the lane) vs relative amount of OCT4 and SOX2 protein is shown. Error bars represent mean of replicates ± standard deviation. The gray horizontal line represents the average % DNA bound by OCT4-SOX2 dimers to the OS site from the Nanog enhancer. (TIF) [file pone.0311120.s003.tif]

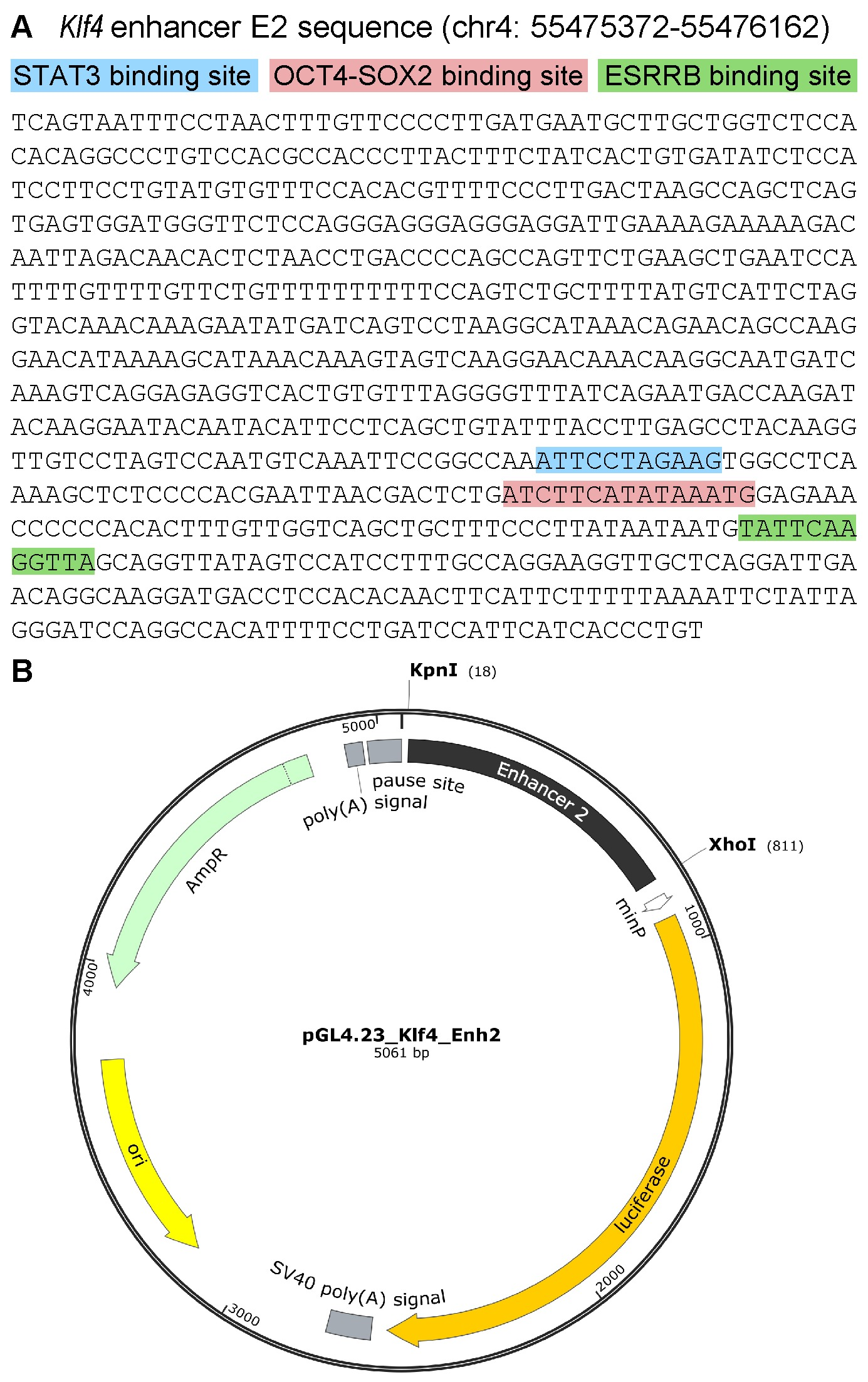

Supplement: S4 Fig — (A) Full sequence of the Klf4 enhancer E2, with sites highlighted for the OCT4-SOX2 composite motif, ESRRB binding site, and STAT3 binding site. (B) Schematic diagram of the construct containing the Klf4 enhancer E2 for luciferase reporter assays. Enhancer E2 was inserted into pGL4.23 at the KpnI and XhoI cut sites. (TIF) [file pone.0311120.s004.tif]

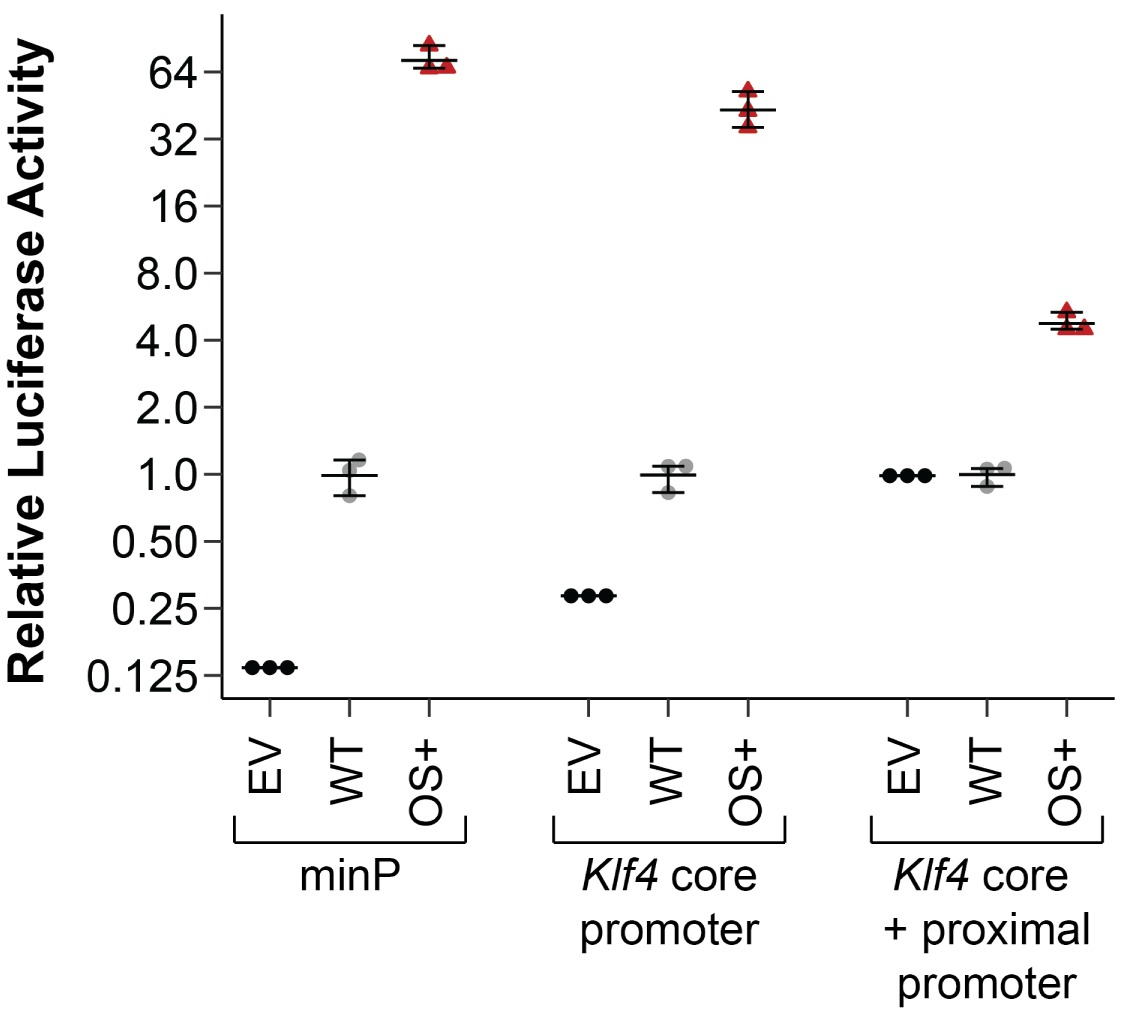

Supplement: S5 Fig — The wild-type (WT) Klf4 enhancer E2 or the high-affinity OCT4-SOX2 substitution mutant (OS+, see also Fig 3A) was cloned into a firefly luciferase reporter plasmid. The minimal promoter (minP) was subsequently replaced with the Klf4 core promoter (-50 to +100, relative to the transcription start site) or the Klf4 core and proximal promoters (-1000 to +100). All constructs were utilized in dual luciferase reporter assays in mESCs. Firefly luciferase reporter data was normalized to Renilla luciferase and then to the empty vector, lacking the Klf4 enhancer E2 sequence (EV). Error bars represent mean of biological replicates ± 95% confidence interval. (TIF) [file pone.0311120.s005.tif]

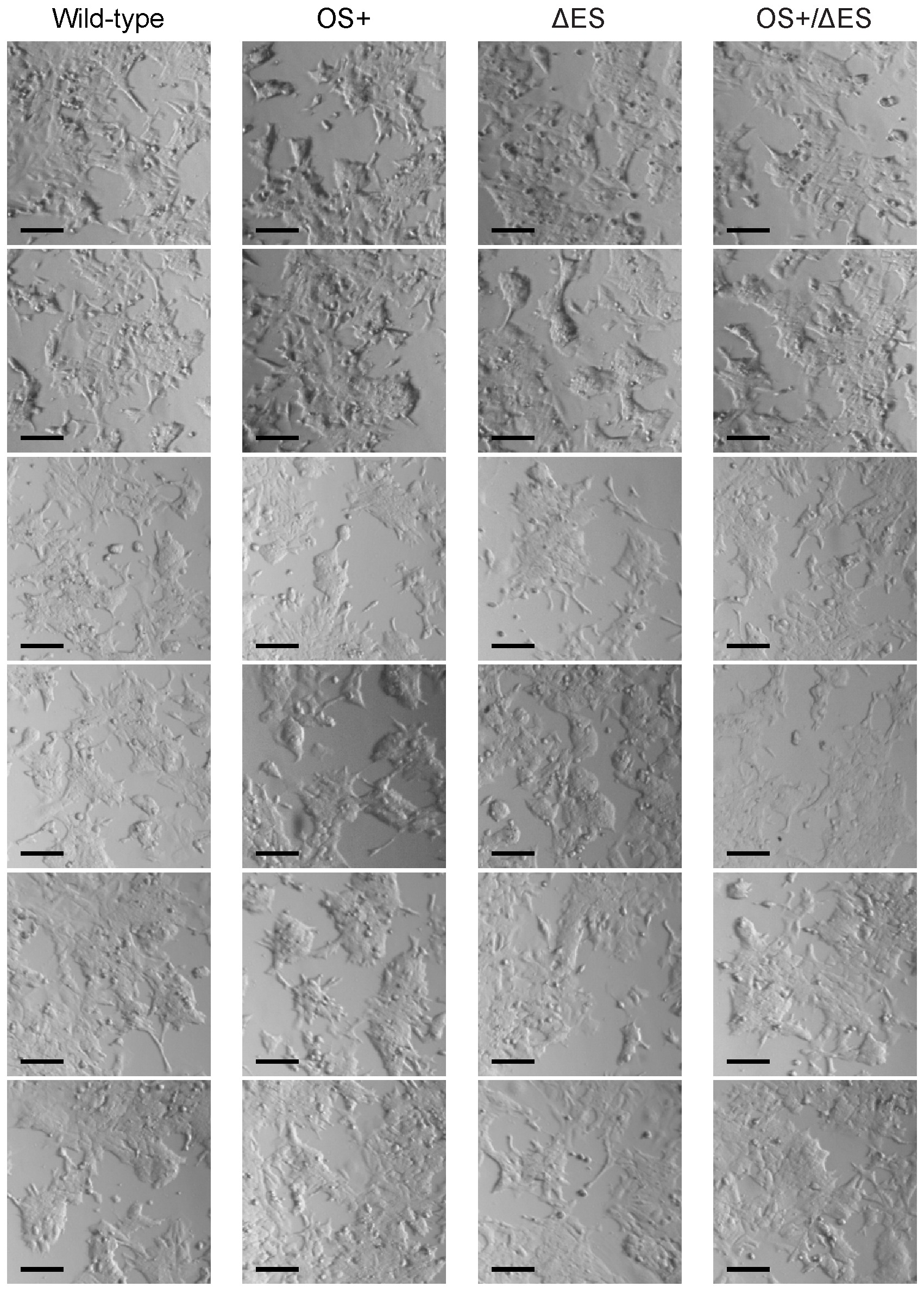

Supplement: S6 Fig — mESCs with endogenous enhancer E2 mutations from Fig 4 were grown on gelatinized 6-well plates and imaged by bright-field microscopy. Each image is of a different clone for the enhancer E2 mutation, which is indicated at the top of the figure. Scale bar represents 100 μm. (TIF) [file pone.0311120.s006.tif]

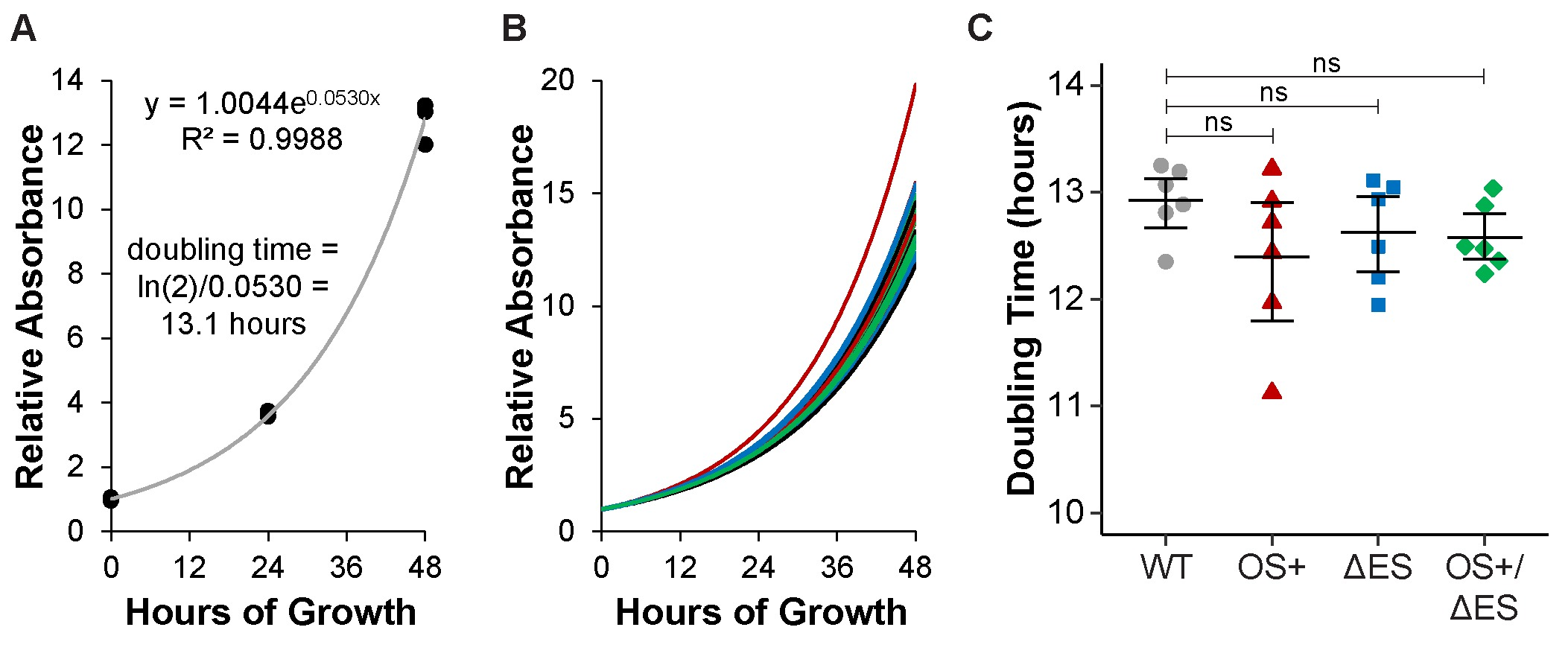

Supplement: S7 Fig — Assays to measure growth rates were performed on mESCs with endogenous enhancer E2 mutations from Fig 4. 10,000 cells were seeded per well in gelatinized 96-well plates, and viable cells were quantified by CCK-8 colorimetric assays at 0, 24, and 48 hours after seeding. Three technical replicates were performed for each clone at each time point. (A) Graph of absorbance over time from one wild-type enhancer E2 clone, with fitted exponential curve and calculations for doubling time. (B) Fitted exponential curves from all clones assayed. (C) Comparison of doubling rates for wild-type and mutant enhancer E2 cell lines. Error bars represent mean of clones ± 95% confidence interval. Unpaired t-tests were performed, and ns signifies not significant. (TIF) [file pone.0311120.s007.tif]

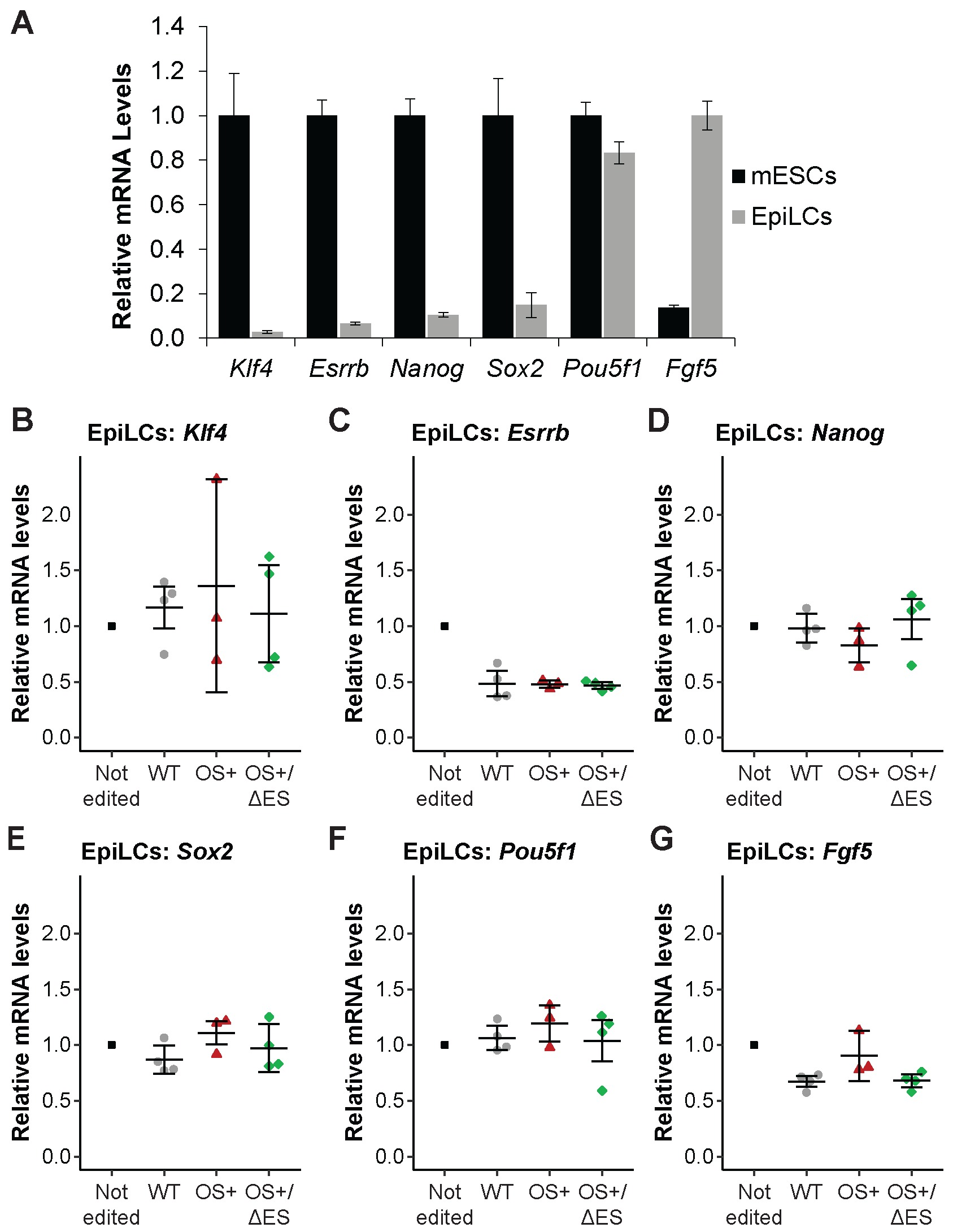

Supplement: S8 Fig — (A) Wild-type mESCs were converted to epiblast-like cells (EpiLCs) by growth in primed-state induction media. Samples of total RNA were collected after 48 hours of induction from EpiLCs and from mESCs, which were grown in standard mESC growth media to maintain naïve-state. Total RNA was reversed transcribed into cDNA for analysis by quantitative PCR. Data was analyzed using the ΔΔCT method. Bars represent mean ± standard deviation (n = 3 technical replicates). (B-G) Comparison of gene expression in EpiLCs generated from unedited, non-RMCE-generated wild-type mESCs and from mESCs containing enhancer E2 mutations from Fig 4. cDNA was synthesized from total RNA collected after 48 hours EpiLC induction, and quantitative PCR was performed to measure levels of Klf4 (B), Esrrb (C), Nanog (D), Sox2 (E), Pou5f1 (F), and Fgf5 (G). Data was analyzed using the ΔΔCT method and normalized to unedited cells. Each data point represents the average from 2–3 technical replicates for each cell line, and error bars represent mean of clones ± 95% confidence interval. (TIF) [file pone.0311120.s008.tif]

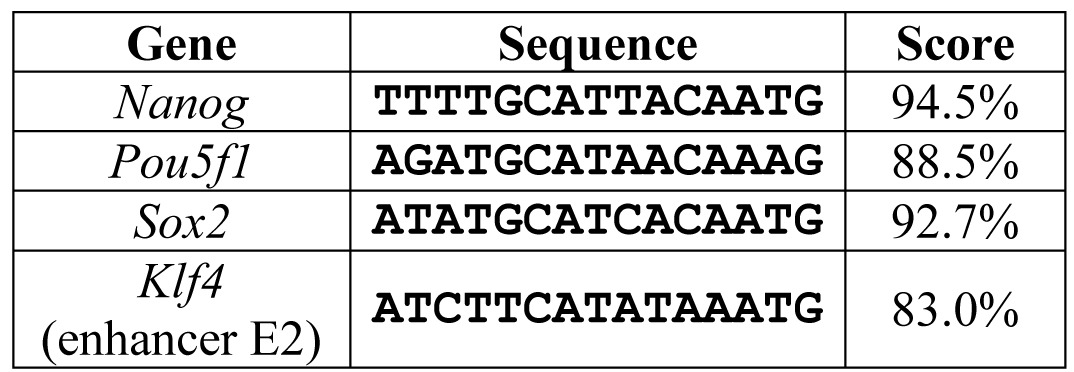

Supplement: S9 Fig — Sequences for OCT4-SOX2 composite binding sites from enhancers for Nanog, Oct4, Sox2, and Klf4 (enhancer E2) were obtained from published papers [28, 37, 38, 75]. The score is the relative score, as calculated on the JASPAR database, using position frequency matrix MA0142.1 [36]. (TIF) [file pone.0311120.s009.tif]
