# Supplementary material for: A suboptimal OCT4-SOX2 binding site facilitates the naïve-state specific function of a Klf4 enhancer
Source: PLoS One. 2024 Sep 30;19(9):e0311120. doi: 10.1371/journal.pone.0311120 (PMC11441684; doi:10.1371/journal.pone.0311120)
Supplement: S1 Table — (DOCX) [file pone.0311120.s010.docx]

**S1 Table. Primers for luciferase reporter assay constructs**

| **Constructs** | **Section** | **Primer sequences (5' to 3')** | |
| --- | --- | --- | --- |
| Oligonucleotides for Inserting OCT4-SOX2 Binding Sites into pGL4.23 | *Nanog* | GTACCAGCTACTTTTGCATTACAATGGCCTTGC | TCGAGCAAGGCCATTGTAATGCAAAAGTAGCTG |
|  | *Klf4*-E1 | GTACCAGCTACCTTTGCATATCAAATGCCTTGC | TCGAGCAAGGCATTTGATATGCAAAGGTAGCTG |
|  | *Klf4*-E2 | GTACCAGCTACAATCTTCATATAAATGGCTTGC | TCGAGCAAGCCATTTATATGAAGATTGTAGCTG |
|  | *Klf4*-E3 | GTACCAGCTACAATTGCATAAAAACAGCCTTGC | TCGAGCAAGGCTGTTTTTATGCAATTGTAGCTG |
| Inserting the *Klf4* Enhancer E2 or promoters into pGL4.23 or pGL4.10 | *Klf4*-E2 | AAAGGAGGTACCTCAGTAATTTCCTAACTTTGTTCCCCTTGATGAATG | AAACCGCTCGAGACAGGGTGATGAATGGATCAGGAAAATGTG |
|  | *Klf4* core promoter | GTCACGAAGCTTCGGGGAGGGGCACTCGGCGG | CTGAGTGATATCGAGCAAGCGAGCGAGAAGTTATAAG |
|  | *Klf4* core and proximal promoter | GTCACGAAGCTTCGGGGAGGGGCACTCGGCGG | CTGAGTGATATCACCTCCCAGTGAAGTCCCTTTGAG |
| Mutating the *Klf4* Enhancer E2 Sequences into pGL4.23 | Optimize OCT4-SOX2 | CGACTCTGTTTTGCATTACAATGGAGAAACCCCCCACACTTTGTTGG | GGGTTTCTCCATTGTAATGCAAAACAGAGTCGTTAATTCGTGGGGAGAG |
|  | Loss of ESRRB | CCCTTATAATAATGTATTCGCCCTTCGCAGGTTATAGTCCATCCTTTGCC | GGACTATAACCTGCGAAGGGCGAATACATTATTATAAGGGAAAGCAGC |
|  | Loss of STAT3 | GGCCAAAATGATACGGGTGGCCTCAAAAGCTCTCCCCACG | GGCCACCCGTATCATTTTGGCCGGAATTTGACATTGGACTAGGAC |
|  | External primers for overlap extension PCR | CGATAGTACTAACATACGCTCTCCATC | GCGCTGGGCCCTTCTTAATG |
