# Supplementary material for: A suboptimal OCT4-SOX2 binding site facilitates the naïve-state specific function of a Klf4 enhancer
Source: PLoS One. 2024 Sep 30;19(9):e0311120. doi: 10.1371/journal.pone.0311120 (PMC11441684; doi:10.1371/journal.pone.0311120)
Supplement: S2 Table — (DOCX) [file pone.0311120.s011.docx]

**S2 Table. Oligonucleotides for EMSAs**

| **Name** | **Sequence (5' to 3')** |
| --- | --- |
| Cy5-labeled primer | CCAGTCTCACCAAGGC |
| *Nanog* | GTTACTCTGCAGCTACTTTTGCATTACAATGGCCTTGGTGAGACTGG |
| *Klf4*-E1 | GTTACTCTGCAGCTACCTTTGCATATCAAATGCCTTGGTGAGACTGG |
| *Klf4*-E2 | GTTACTCTGCAGCTACATCTTCATATAAATGGCCTTGGTGAGACTGG |
| *Klf4*-E3 | GTTACTCTGCAGCTACAATTGCATAAAAACAGCCTTGGTGAGACTGG |
