# Supplementary material for: A suboptimal OCT4-SOX2 binding site facilitates the naïve-state specific function of a Klf4 enhancer
Source: PLoS One. 2024 Sep 30;19(9):e0311120. doi: 10.1371/journal.pone.0311120 (PMC11441684; doi:10.1371/journal.pone.0311120)
Supplement: S3 Table — (DOCX) [file pone.0311120.s012.docx]

**S3 Table. Primers for gene editing constructs**

| **Plasmid constructed** | **Section of the plasmid** | **Primer sequences (5' to 3')** | |
| --- | --- | --- | --- |
| Oligonucleotides to insert sgRNA sequences into the Cas9 expression plasmid | sgRNA #1 | CACCGTCATCAAGGGGAACAAAGTT | AAACAACTTTGTTCCCCTTGATGAC |
|  | sgRNA #2 | CACCGCAATCCAGCTACTATAGTTT | AAACAAACTATAGTAGCTGGATTGC |
| Primers to construct the HDR Template for the *Klf4*-FRT-mCherry-E2-F3 mESC line | *Klf4* enhancer E2 | CATGCACTCGAGTCAGTAATTTGCTAACTTTGTTCCCCTTGATGAATGC | CATGCACCGCGGGCAAAACTATAGTAGCTGGATTGGTGATACATG |
|  | mCherry expression cassette | GGCCTAACTGGCCGGTACC | ATCTAGCCGCGGTAAGATACATTGATGAGTTTGGACAAACCAC |
|  | Upstream Homology Arm | ACGTCAGGTACCGGATGAGTTGATAGAGGAATGGATAGCCG | TAATATTGATACATATACAATGATACAAATGTATCAAAAGAAGCC |
|  | Downstream Homology Arm | AAATTGCTATTTTATTGTATGCTCAGCGGC | CATGACGTCGACATGCAGCACCGCTGACTAAAGTAATTCTTG |
| Primers to construct the recombinase-mediated cassette exchange plasmid | *Klf4* enhancer E2 | TCAGTAATTTCCTAACTTTGTTCCCCTTGATGAATGC | CCAAAACTATAGTAGCTGGATTGGTGATACATGC |
