# Supplementary material for: A suboptimal OCT4-SOX2 binding site facilitates the naïve-state specific function of a Klf4 enhancer
Source: PLoS One. 2024 Sep 30;19(9):e0311120. doi: 10.1371/journal.pone.0311120 (PMC11441684; doi:10.1371/journal.pone.0311120)
Supplement: S4 Table — (DOCX) [file pone.0311120.s013.docx]

**S4 Table. Primers for genotyping gene edited mESCs**

| **Purpose** | | **Primer sequences (5' to 3')** |
| --- | --- | --- |
| Screening the *Klf4*-FRT-mCherry-E2-F3 mESC Line | Integration near the upstream homology arm | AATCAGTGACTGGGTGGAGAAATAGGC;  AAGTAAGGGTGGCGTGGACAG;  AGAAAGCGAAGGAGCAAAGCTGC |
|  | Integration near the downstream homology arm | CCTGTATCCCAAACAGAGCTCCG; CCAATCCAGCTACTATAGTTTTGGAAATTGC; CTATAGTTTTGCCCGCGGTGG |
| Screening after recombinase-mediated cassette exchange | | AATCAGTGACTGGGTGGAGAAATAGGC; AAGTAAGGGTGGCGTGGACAG; GGAACAAAGTTAGGAAATTACTGACGAAGTTCC |
| Primers for automated Sanger sequencing | | ACTGCCTTGGGAAAAGC;  CATGACGTCGACATGCAGCACCGCTGACTAAAGTAATTCTTG;  ACAGGGTGATGAATGGATCAGGAAAATGTGG |
