# Supplementary material for: A suboptimal OCT4-SOX2 binding site facilitates the naïve-state specific function of a Klf4 enhancer
Source: PLoS One. 2024 Sep 30;19(9):e0311120. doi: 10.1371/journal.pone.0311120 (PMC11441684; doi:10.1371/journal.pone.0311120)
Supplement: S5 Table — (DOCX) [file pone.0311120.s014.docx]

**S5 Table. Primers for RT-qPCR**

| **Gene** | **Forward Primer (5' to 3')** | **Reverse Primer (5' to 3')** |
| --- | --- | --- |
| *Esrrb* | GCACCTGGGCTCTAGTTGC | TACAGTCCTCGTAGCTCTTGC |
| *Fgf5* | AAGTAGCGCGACGTTTTCTTC | CTGGAAACTGCTATGTTCCGAG |
| *Gapdh* | AGGTCGGTGTGAACGGATTTG | TGTAGACCATGTAGTTGAGGTCA |
| *Klf2* | CTCAGCGAGCCTATCTTGCC | TGTTTAGGTCCTCATCCGTGC |
| *Klf4* | GTGCCCCGACTAACCGTTG | GTCGTTGAACTCCTCGGTCT |
| *Klf5* | AGCGACGTATCCACTTCTGC | CAGGTGCACTTGTAGGGCTT |
| *Nanog* | TCTTCCTGGTCCCCACAGTTT | GCAAGAATAGTTCTCGGGATGAA |
| *Pou5f1* | CCAGGCAGGAGCACGAGTGG | CCTGGGACTCCTCGGGAGTTG |
| *Rad23b* | ACCTTCAAGATCGACATCGACC | ACTTCTGACCTGCTACCGGAA |
| *Sox2* | GCGGAGTGGAAACTTTTGTCC | CGGGAAGCGTGTACTTATCCTT |
| *Tbx3* | GAGGCCAAGGAACTTTGGGA | TATCGACAGTCGTCAGCAGC |
