# Supplementary material for: A suboptimal OCT4-SOX2 binding site facilitates the naïve-state specific function of a Klf4 enhancer
Source: PLoS One. 2024 Sep 30;19(9):e0311120. doi: 10.1371/journal.pone.0311120 (PMC11441684; doi:10.1371/journal.pone.0311120)
Supplement: S1 Raw images — (PDF) [file pone.0311120.s016.pdf]

Raw image for Figure 2A

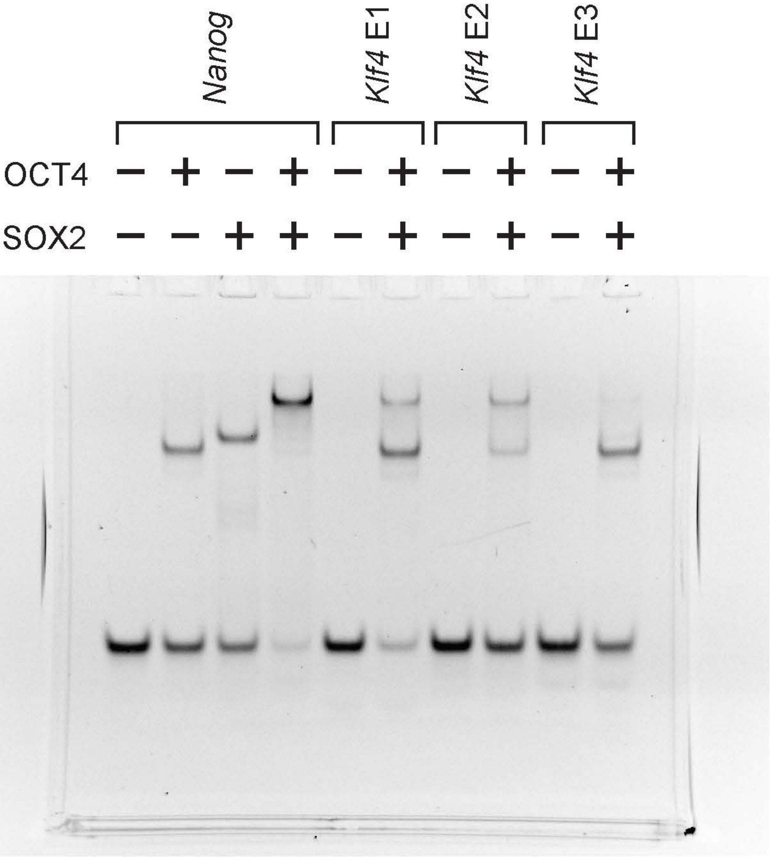

Method to capture the image:
DNA probes were end labeled with Cy5 and detected after gel electrophoresis on a Bio-Rad ChemiDoc XRS+, using the Cy5 channel, with a 30 second exposure. Image was exported as a tif file.

Molecular weight markers are not utilized in gel shift assays.

Raw image for Figure S3A

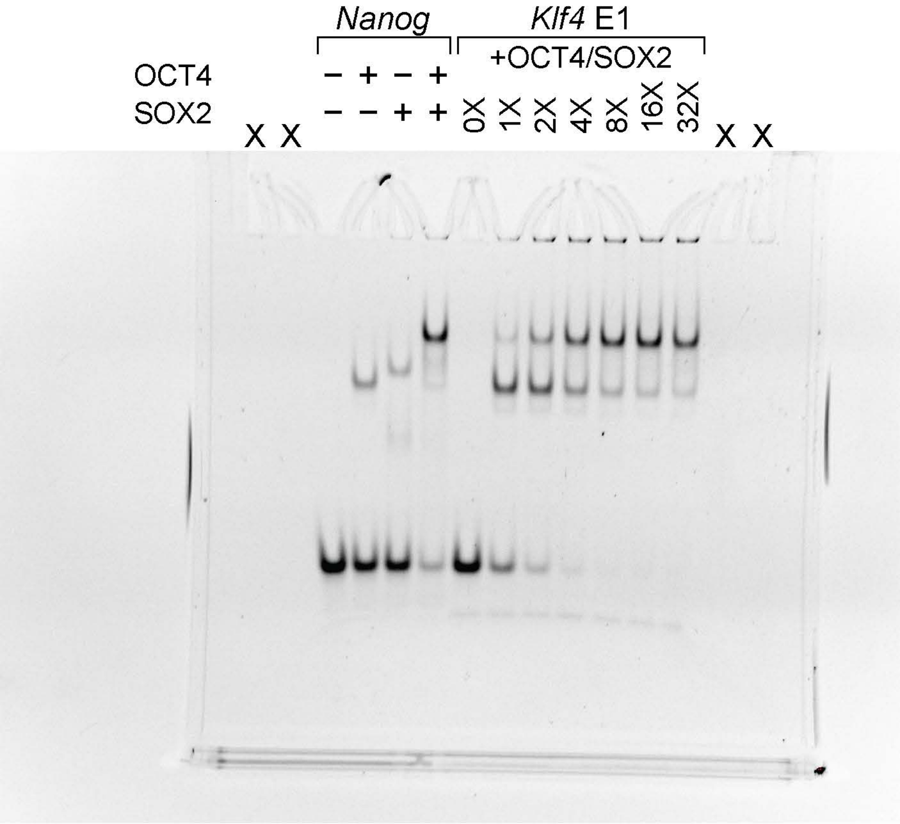

Method to capture the image:
DNA probes were end labeled with Cy5 and detected after gel electrophoresis on a Bio-Rad ChemiDoc XRS+, using the Cy5 channel, with a 30 second exposure. Image was exported as a tif file.

Molecular weight markers are not utilized in gel shift assays.

Raw image for Figure S3C

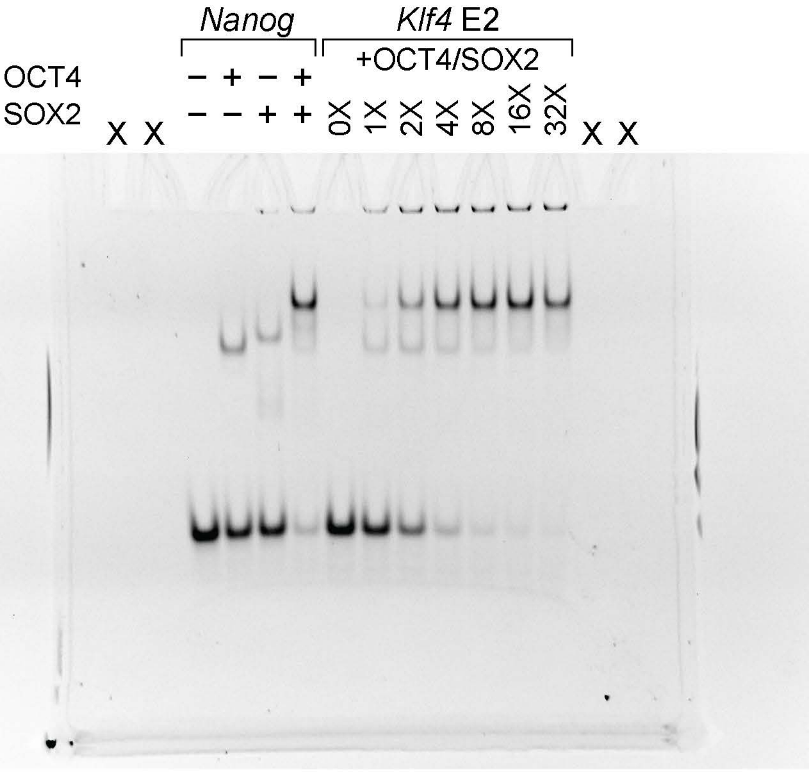

Method to capture the image:
DNA probes were end labeled with Cy5 and detected after gel electrophoresis on a Bio-Rad ChemiDoc XRS+, using the Cy5 channel, with a 30 second exposure. Image was exported as a tif file.

Molecular weight markers are not utilized in gel shift assays.

Raw image for Figure S3E

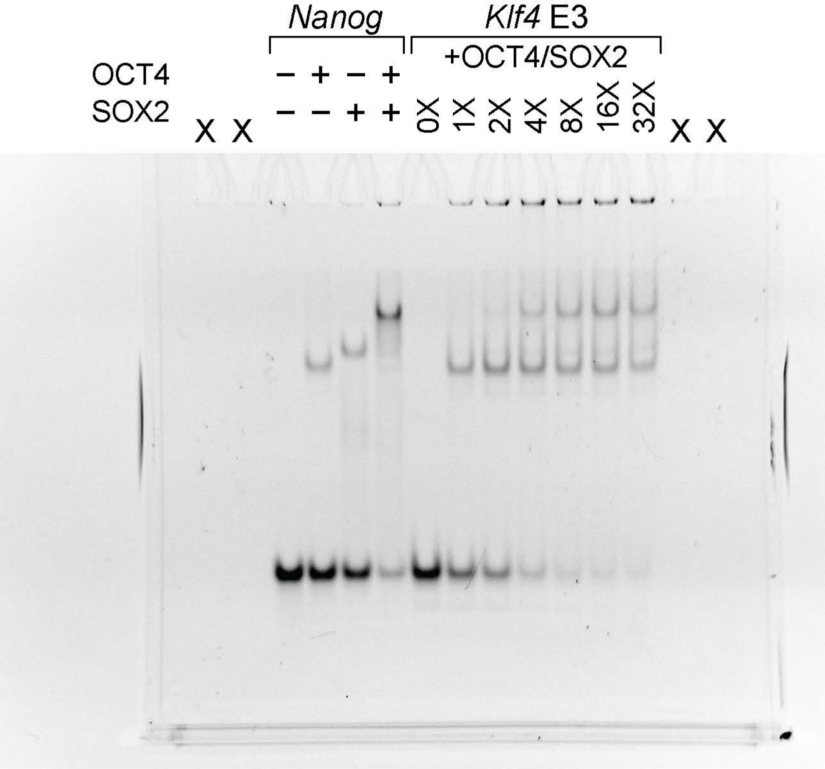

Method to capture the image:
DNA probes were end labeled with Cy5 and detected after gel electrophoresis on a Bio-Rad ChemiDoc XRS+, using the Cy5 channel, with a 30 second exposure. Image was exported as a tif file.

Molecular weight markers are not utilized in gel shift assays.
