# Supplementary material for: A suboptimal OCT4-SOX2 binding site facilitates the naïve-state specific function of a Klf4 enhancer
Source: PLoS One. 2024 Sep 30;19(9):e0311120. doi: 10.1371/journal.pone.0311120 (PMC11441684; doi:10.1371/journal.pone.0311120)
Supplement: Attachment — Submitted filename: Response to Reviewers.pdf [file pone.0311120.s017.pdf]

<u>**Response to Reviewers**</u>
PONE-D-24-04969

Authors' responses to reviewer comments are in blue italics. References to pages and lines in the text correspond to the manuscript without tracked changes.

Reviewer #1: Enhancers are well known to play pivotal roles in transcriptional regulation. In this study, the authors sought to investigate the enhancer activity of a naïve-specific gene, Klf4, in mouse ESCs. In order to assess the enhancer activities, they have performed gel shift assay, luciferase assay, and recombinase-mediated cassette exchange. As a result, in the Klf4 enhancer region, the authors identified low-affinity OCT4-SOX2 binding sites, which could explain the different transcriptional regulation between naïve and primed pluripotent states. This study is potentially interesting because it might provide new insights into the transcriptional regulation of a naïve-specific gene, Klf4. However, the authors should address the following points to ensure the accuracy of their study and strengthen their arguments before this manuscript could be considered for publication in PLOS ONE.

A Major comment
The main concern is the degree of involvement of low- or high-affinity OCT4-SOX2 binding site for Klf4 expression in mESCs. Their luciferase assay in EpiLCs showed that high-affinity OCT4-SOX2 binding site (OS+) had higher enhancer activity (Fig. 5). However, OS+ endogenous substitution in EpiLCs did not upregulate Klf4 expression (Fig. S5). What made the discrepancy? It is not clear whether the enhancer functions in the actual genome. This point should be carefully interpreted and discussed.

> *We thank Reviewer #1 for this question and suggestion. In the manuscript text (see page 18, lines 380-393), we have now expanded our discussion of our data to address questions about the discrepancy between the luciferase reporter assays and the mutant EpiLCs.*

Other comments
1.The authors mainly investigated the enhancers for Klf4. To ensure clarity for the readers, the positional information, such as the distance between the enhancers (E1, E2, and E3) and the Klf4 TSS site, should be illustrated.

> *We agree with Reviewer #1 that this would be informative and helpful to readers. The NEW S2 Figure includes a diagram to map the enhancers and TSS for* Klf4*.*

2.The authors mainly analyzed a limited number of gene expressions by qPCR in WT and mutant cells. Other phenotypes, including the colony appearance and cell proliferation potential, should be presented.

> *We thank Reviewer #1 for these suggestions, as further analysis of our wild-type and mutant cell lines would strengthen our study. We have now performed more qPCR measurements (REVISED Figures 4 and 5), included images of colony morphology (NEW S6 Figure), and added growth assay data assessing cell proliferation potential (NEW S7 Figure). Specifics for these additional experiments are described below, in responses to other points by Reviewer #1.*

3.In Figure 4, the authors should examine expression levels of other naïve-specific genes, such as Esrrb, Tbx3, or Dppa3, or Zfp42. Also, the colony appearance of ESCs should be presented.

The authors should confirm thatΔES and OS+/ΔES-cells show flattened or dome-shaped colonies.

We have addressed this comment by performing new qPCR analyses to measure levels of the naïve-state specific genes Esrrb *and* Tbx3 *for our wild-type and mutant cell-lines in the naïve-state. These data are included in the REVISED Figure 4. We also have characterized and included other phenotypes. We have added images for colony appearance for our wild-type and mutant cell lines, which are shown in NEW S6 Figure.*

4.In Figure 4 and S5, for the naïve to formative transition experiment in enhancer-substituted cells, the authors should compare naïve and pluripotent gene expression before and after the transition.

To address this important point, we have now included additional, new qPCR analyses for Pou5f1, Sox2, Nanog, Esrrb, *and* Tbx3 *for our wild-type and mutant cell-lines in the induced formative-state EpiLCs. We also have added qPCR results for these cell-lines before induction. These are included in the REVISED Figure 5 (Figure S5 in the original submission).*

5.Previous studies showed that Klf family members, including Klf2, 4, and 5, work in concert with other family members to facilitate self-renewal or maintain the undifferentiated state (Nat Cell Biol. 2008;10(3):353-60, Nat Commun. 2014;5:3719). How about the expression of other Klf family members in enhancer-substituted cells? Also, is there any difference in their proliferation potential?

While KLF2, KLF4, and KLF5 form a circuit to promote naïve-state pluripotency and self-renewal, prior studies have not indicated that they regulate each other. However, Klf2 *and* Klf5 *appear to be naïve-state specific. So, we have performed new qPCR analyses to measure levels of* Klf2 *and* Klf5 *in our mutant cell-lines. These are included in the REVISED Figure 4.*

We have also performed new cell growth assays to assess cell proliferation potential on our mutant cell lines. These data are presented in the NEW S7 Figure.

Reviewer #2: Torigoe and colleagues use the composite SoxOct element to study the DNA motif grammar associated with pluripotency using a specific example. They focus on the auto-regulation of pluripotency genes and examine how Sox/Oct4 drive Klf4 expression in pluripotent states in vitro using putative Sox2/Oct4 binding sites. This follows on an GnD paper from 2017 where the same enhancer elements were studied. They perform luciferase assays, EMSAs and studies in mouse ESCs in pre- and post-implantation culture conditions. EMSAs and luciferase assays suggest that cooperative binding and gene activation is weaker in the three KLF4 enhancer. They then further analyse site E2 that this critical for Klf4 activation despite the reduced dimer formation using luciferase assays and RCME. The main takeaway of the study is that degenerate binding sites are important for context-dependent gene regulation. Whilst this is not new per se it's interesting to show this for this enhancer element. The advance of the study is incremental but of interest to those with a focus on gene regulation in ESCs and the role of different cis-regulatory elements.

General comments:

• The introduction should elaborate on alternative SoxOct binding sites such as the compressed motif targeted by SOX17/OCT4 and the FGF4 SoxOct element with 3bp spacer

We thank Reviewer #2 for bringing this to our attention. We have added discussion of alternative oct-sox motifs into the beginning of Results (see pages 4 and 5, lines 77-88), when we initially discuss our observations about the oct-sox motifs in the Klf4 *enhancers.*

• EMSAs in Figure 2, and elsewhere are of good quality and could be used to calculate the cooperativity factor omega (all four microstates would need to be quantified using densitometry). The study would benefit from a quantitative analysis of the binding affinity or cooperativity (authors use the term affinity a lot without quantitation). Related 'sub-optimal' motifs might have been studied elsewhere and may help interpretation (i.e. https://doi.org/10.1093/nar/gkw1198). The overall claim that cooperative binding is Nanog>E1>E2>E3 looks convincing. Ideally, all experiments should be performed in triplicates, monomer Sox and Oct lanes should be included and quantitation provided. I realize that the Sox2 band is a bit weak in most lanes where two proteins are present. If estimations of omega are not possible for the weak Sox lane other quantitative comparisons could be considered (i.e. plotting the dimer fraction for the various DNA probes normalized by a control). In methods, please provide more details for EMSAs. Rather than the quantities of proteins provide molar concentrations for all reactants. Are these full-length proteins expressed in E.coli? Authors refer to ref28 but key points should be reported here.

We thank Reviewer #2 for their comments and suggestions on our EMSA experiments. Reduced cooperative binding by OCT4 and SOX2 at the Klf4 *enhancers is one potential interpretation of our data, requiring calculation of the cooperativity factor omega for each sequence. Unfortunately, as also noted by Reviewer #2, the SOX2-DNA band being undetectable in most cases, so we are unable to confidently quantify monomer binding and, hence, calculate cooperativity factor omega.*

Nevertheless, we agree that quantification of our EMSA results would strengthen our conclusions about differences in binding affinity of the OCT4-SOX2 dimer to our DNA probes. We have calculated the percentage of DNA bound by the OCT4-SOX2 dimer, which is normalized to the overall Cy5 signal in each lane. These data have been added to REVISED Figure 2 and S3 Figure (formerly S2 Figure).

We also thank Reviewer #2 for their reference to the Chang, et al, 2017, Nucleic Acids Research paper. In that study, the authors examined the effects of spacing, orientation and some sequence alterations on cooperative binding by OCT and SOX proteins, including OCT4 and SOX2. While their sequence analyses do not include the differences we observe in the OCT4-SOX2 sites from the Klf4 *enhancers, this study emphasizes that OCT4 and SOX2 cooperative binding is significantly impacted by the spacing and relative orientations of the individual OCT4 and SOX2 binding sites. Based on this study, the OCT4-SOX2 sites in the* Klf4 *enhancers have the optimal spacing and relative orientation. We have revised our text to discuss this study and its relevance to the OCT4-SOX2 sites from the* Klf4 *enhancers (see pages 4 and 5, lines 80-83).*

We also would like to note that, as requested by Reviewer #2, we had done our EMSAs at least in triplicate prior to the original submission, as noted in the figure legends. We apologize if this information was not made more clear in the original submission. We

have added a note about replicates into the Methods for EMSAs (see page 26, lines 560-561).

Lastly, we agree that more information about the EMSAs would be useful. Our revised Methods now contain more complete information about our protocols (see pages 24-26; lines 529-570). Where appropriate, we also converted to molar concentrations, namely for OCT4, SOX2 and the Cy5-labeled probes. For other reagents, concentrations are provided in units according to common practice.

• Suggest to show a UCSC ChIP-seq signal genome browser track for E1, E2, E3. This could easily be obtained from public ChIP-seq data sets (i.e. https://compbio-zhanglab.org/CRCistrome/index.php). I realise some of this is shown in ref28.

We agree with Reviewer #2 that showing genome browser tracks from ChIP-seq data would reiterate that OCT4, SOX2, ESRRB, and STAT3 bind to the Klf4 *enhancers. We have generated NEW S2 Figure with genome browser tracks for all four of these transcription factors.*

• Data in Figure 3 are interesting but could be explained more clearly in the results. Please show the sequence and indicate the length of the reporter construct in Figure 3. Here and elsewhere make clear which cells were analysed (same in Figure 5).

We have revised the text, in which we discuss Figure 3 (see pages 10 and 11, lines 196-219), and we hope that the revised explanations are more clear for readers.

We agree that it would be helpful for readers to have further information about our mutant enhancer E2 sequences and the reporter constructs. We have made the following changes to our manuscript:

- *We have added the chromosome coordinates for enhancer E2 into our Methods, similar to other genomic DNA coordinates that we also provided. We apologize for their omission in the original submission, as those would be useful to readers.*
- *We have generated NEW S4 Figure, which includes (A) the entire* Klf4 *enhancer E2 sequence used in the luciferase reporter assays, with relevant TF binding sites labeled and (B) a schematic map of the constructs for luciferase reporter assays with* Klf4 *enhancer E2.*
- *The REVISED Figure 3A now displays sequences for the relevant TF binding sites, as well as the mutations we utilized in our study.*

We hope that these revisions address the questions from Reviewer #2 about the sequences and constructs used in Figure 3.

To all figures with data from mESCs or EpiLCs, we have now added notations onto the figures to more clearly designate which cells were being analyzed.

• Were the RCME-based reporter assays performed in both naïve ESCs and EpiLCs? Suggest showing EpiLC and naïve reporter data for the enhancer mutants side by side to back up the key claim that the motif degeneracy is the key for the specific expression of Klf4 in naïve ESCs.

All luciferase reporter assays and qPCR analyses of RMCE-generated cell lines were performed in both mESCs and EpiLCs. As noted with the previous comment, we have

now added notations onto figures to clearly indicate the cell-type utilized in each experiment.

We thank Reviewer #2 for the suggestion to show naïve-state and primed-state data side-by-side, as this will more effectively demonstrate the naïve-state specific function of Klf4 *enhancer E2. We have re-configured Figures 3 and 5 into a single figure, putting all the luciferase reporter data together into REVISED Figure 3. In the REVISED Figure 5 (formerly S5 Figure), qPCR analyses of our RMCE-generated cell-lines from both before and after induction to EpiLCs are shown side-by-side.*

- Are the enhancers E1-E3 conserved in humans?

We thank Reviewer #2 for this excellent question, as we also have wondered about the conservation of the Klf4 *enhancers across species, particularly in humans. We have utilized data from ENCODE to identify candidate enhancers for* Klf4 *in humans. We found one possible sequence in a similar location as E1 and E2 (about 50 kb downstream of the TSS) that had predicted binding sites for OCT4, SOX2, ESRRB, and STAT3. Analysis of OCT4 ChIP-seq data from human ESCs revealed a peak at this location, and the OCT4-SOX2 composite site appears to be suboptimal, based on initial scans with JASPAR. While promising, further experimentation is required to confirm functionality of this enhancer in human ESCs, which is beyond the scope of the current manuscript.*
