# Supplementary material for: A suboptimal OCT4-SOX2 binding site facilitates the naïve-state specific function of a Klf4 enhancer
Source: PLoS One. 2024 Sep 30;19(9):e0311120. doi: 10.1371/journal.pone.0311120 (PMC11441684; doi:10.1371/journal.pone.0311120)
Supplement: Attachment — Submitted filename: Response to Reviewers 2.pdf [file pone.0311120.s018.pdf]

Response to Reviewers
PONE-D-24-04969R1

Authors' responses are in blue italics

Reviewer #1: Although the impact of low- and high-affinity Oct4-Sox2 binding site on the expression of Klf4 or the functional regulation of pluripotency, such as other naïve-related gene expressions and colony appearance, is not highly significant, their additional experiments clarified the claims in the manuscript. As the authors discussed, further studies will be required to understand the mechanisms involved in the enhancer activity. Their revised manuscript is now worth considering for publication in PLOS One.

We thank Reviewer #1 for their review of our revised manuscript. We appreciate that they find our additional experiments clarifying of our conclusions, such that the manuscript meets expectations for publication.

My additional minor comments on the manuscript are as follows:

Minor comments:
1. Line 376-393: These discussions should be included in the Discussion section.

We have moved these to the Discussion section and have edited the text accordingly.

2. Line 377 and 380: Fig 5B -> Fig 5A

We apologize for the typos in reference to Figure 5. We have corrected these in the manuscript.

Reviewer #2: The authors have addressed all my comments and I am supportive of accepting this nice work. I'd encourage authors to ensure that statistical tests and replications of experiments follow rigorous standards and that plasmids and raw data are made available in public repositories (figshare, add gene) if appropriate.

We thank Reviewer #2 for their review of our revised manuscript, and we are glad to hear that we have addressed all of their comments. We appreciate their recommendation to accept our manuscript for publication.

We have made a few minor edits to the Methods section to add more details to clarify our statistical tests and replications of experiments. Some of these details were included in the figure legends, and they are now added to the Methods.

Plasmids have already been deposited to Addgene, and they will be made available when the manuscript is published. All raw quantitative data was included with the revised manuscript as a Supporting Information file (S1_file) to be made available with the paper upon publication. Raw images for EMSAs are already available on the Open Science Framework, and DOI information was provided in the submission information for the revised manuscript.
